# No Stone Left Unturned: A Rare Case of Viridans Strep Bacteremia Secondary to Choledocholithiasis

**DOI:** 10.7759/cureus.84778

**Published:** 2025-05-25

**Authors:** Yasasvhinie Santharam, Parth Desai, Nadim A Qadir, Brian Li, Pramod Reddy

**Affiliations:** 1 Internal Medicine, University of Florida College of Medicine – Jacksonville, Jacksonville, USA

**Keywords:** bacteremia, cbd stone, choledocholithiasis, common bile duct dilatation, viridans group streptococci

## Abstract

When assessing a patient with bacteremia to develop an appropriate treatment plan, it is important to identify the most likely source in order to achieve source control as well as provide adequate antibiotic coverage per culture results. Through confirmation bias and the availability heuristic, it is easy to consider only the most commonly known or first options that come to mind when thinking of the source of a particular type of bacteria, potentially missing the actual source of the infection. The purpose of this report is to highlight an interesting case of undifferentiated viridans group streptococci (VGS) bacteremia where the source was not the oropharynx as is commonly thought, but rather translocation secondary to choledocholithiasis.

## Introduction

Viridans group streptococci (VGS) are a class of bacteria known to cause bloodstream infections particularly in patients with neutropenia, valvular abnormalities, or a history of recent dental procedures [1]. The primary mechanism for VGS bacteremia is translocation into the bloodstream when mucosal barriers are compromised. While the most common source of VGS bacteremia in neutropenic patients is infiltration from the oral mucosa after dental procedures, a source often implicated in non-neutropenic patients with particular strains of VGS bacteria is hepatobiliary infection [2-3]. Here, we present the case of a non-neutropenic 84-year-old woman who was hospitalized for ascending cholangitis and choledocholithiasis complicated by VGS bacteremia. This case is important as it highlights a rare instance of VGS bacteremia and the need for clinicians to consider non-oral gastrointestinal sources when assessing these patients.

## Case presentation

An 84-year-old White woman with a past medical history of heart failure with a reduced ejection fraction of 25%-30%, coronary artery disease followed by percutaneous coronary intervention, ischemic cardiomyopathy with implanted cardioverter/defibrillator placement, and paroxysmal atrial fibrillation but not on anticoagulation due to repeat falls, initially presented to the emergency department (ED) due to two weeks of generalized weakness and fatigue, a fall secondary to orthostatic hypotension, and dizziness, as well as three days of generalized abdominal pain, nausea, and vomiting. Her weakness and fatigue were unchanged with rest or oral hydration protocols, her abdominal pain was only mildly improved with over-the-counter acetaminophen, and her nausea and vomiting were unchanged with ginger chews or lemon water. There were no reported aggravating factors for her symptoms.

The initial physical exam was remarkable for disorientation to location, mild abdominal distention, and diffuse abdominal tenderness. The patient was normotensive with a regular heart rate, but noted to be febrile (102°F). Her laboratory workup was significant for transaminitis with aspartate aminotransferase (AST) levels at 478 IU/L, alanine aminotransferase (ALT) at 294 IU/L, and alkaline phosphatase at 703 IU/L, along with direct hyperbilirubinemia with a total bilirubin level 2.9, lactic acidosis, leukocytosis, and a 25% bandemia (Table 1).

**Table 1:** Lab values on admission

Laboratory tests	Patient's results	Normal range	Value interpretation
White blood cell count	12.49 x 10^9^ cells/L	4-11 x 10^9^ cells/L	Elevated
Percent bands	25.2%	0-10%	Elevated
Percent neutrophils	84.1%	34%-73%	Elevated
Na+	135 mmol/L	135-145 mmol/L	Normal
K+	3.6 mmol/L	3.5-4.5 mmol/L	Normal
H+	104 mmol/L	98-107 mmol/L	Normal
HCO_3_^-^	22 mmol/L	21-29 mmol/L	Normal
Anion gap	9 mmol/L	4-12 mmol/L	Normal
Urea nitrogen (BUN)	17 mg/dL	6-22 mg/dL	Normal
Creatinine (Cr)	1.20 mg/dL	Baseline: 1.0-1.2 mg/dL	Normal
Aspartate aminotransferase (AST)	478 IU/L	14-33 IU/L	Elevated
Alanine aminotransferase (ALT)	294 IU/L	10-42 IU/L	Elevated
Alkaline phosphatase	703 IU/L	35-104 IU/L	Elevated
Total bilirubin	2.9 mg/dL	0.2-1.0 mg/dL	Elevated
Direct bilirubin	2.1 mg/dL	<0.2 mg/dL	Elevated

**Figure 1:**
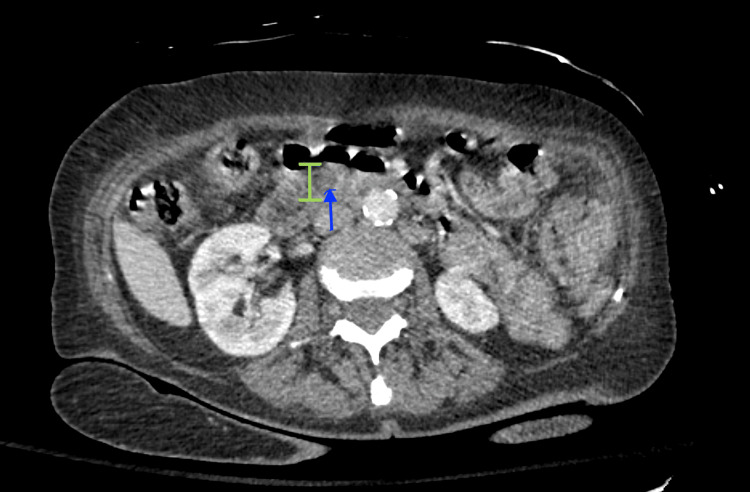
CT scan of the abdomen/pelvis showing increased dilatation of the common bile duct measuring up to 9 mm (green distance marker), and a subtle area of hyperattenuation measuring 5 mm (blue arrow) within the distal common bile duct

A CT scan of the abdomen and pelvis was performed on day of admission (DOA) 1, with findings concerning for choledocholithiasis, with intrahepatic and common bile duct dilatation secondary to a 5-mm stone, acute cholecystitis, gastritis, and diverticulosis (Figure 1).

Given the patient's fever, abdominal pain, elevated bilirubin and liver enzymes, and altered mentation in the setting of imaging-confirmed choledocholithiasis, the patient was transferred from the ED directly to the intensive care unit (ICU) for sepsis secondary to ascending cholangitis.

Two sets of blood cultures drawn 15 minutes apart from two different locations of peripheral veins were collected on DOA 1 as part of a sepsis workup. In the ICU on DOA 1, the patient was initiated on IV piperacillin-tazobactam and IV vancomycin for broad-spectrum coverage. On DOA 2, the cultures returned positive for Streptococcus species, and vancomycin was discontinued as there was no further concern for methicillin-resistant *Staphylococcus aureus* (MRSA) bacteremia. On DOA 3, the initial blood cultures returned positive for VGS, and the Infectious Disease (ID) team was consulted.

On DOA 4, a transthoracic echocardiogram was performed, revealing preserved ejection fraction and diastolic filling, no wall motion abnormalities, and no vegetations. There was some concern initially that the blood cultures were positive for VGS as a contaminant from skin flora, and they thus did not undergo further speciation. However, as they were positive in two sets of cultures and the patient was acutely ill, her cardiac imaging results were negative, she did not have a history of recent dental procedures, and she did not have central lines, the patient’s primary risk factor for VGS bacteremia was deemed to be her acute choledocholithiasis. At this point, the patient was transitioned from piperacillin-tazobactam to IV ceftriaxone.

**Figure 2:**
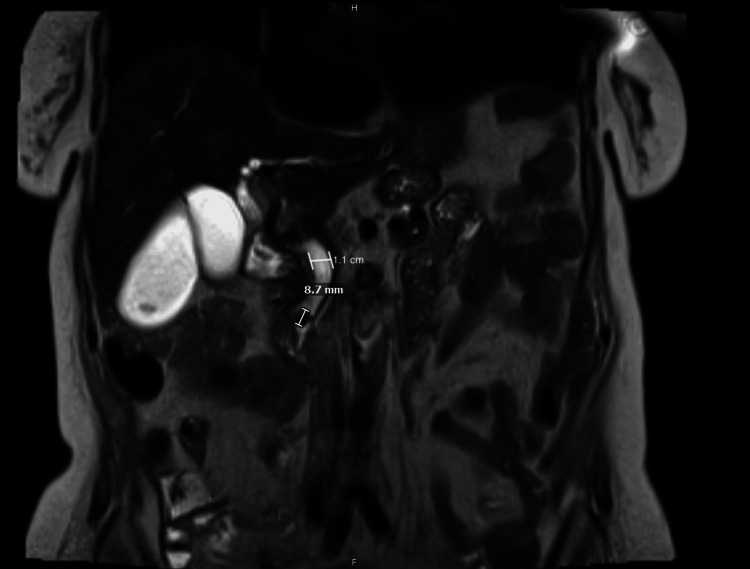
T2-weighted MRCP showing dilation of the intrahepatic and extrahepatic biliary system, with the common bile duct measuring 1.1 cm, and evidence of a distal CBD obstructing stone measuring 8.7 mm MRCP: magnetic resonance cholangiopancreatography; CBD: common bile duct

On DOA 5, magnetic resonance cholangiopancreatography (MRCP) was performed, revealing a 0.87-cm choledocholithiasis within the distal common bile duct with upstream biliary ductal dilation, cholelithiasis, and gallbladder sludge (Figure 2).

Gastroenterology (GI) was consulted, and plans were made for endoscopic ultrasound (EUS) and endoscopic retrograde cholangiopancreatography (ERCP). On DOA 6, repeat blood cultures were collected. On DOA 7, GI performed an EUS and ERCP, with evidence of a 10-mm area of sludge/stone in the gallbladder, as well as a sphincterotomy with sludge removal and a balloon sweep. On DOA 8, as source control was achieved with the removal of the stone and the patient experienced improvement in her symptoms, remaining afebrile with a stable white blood cell count, she was deemed safe for discharge home. At discharge, she was transitioned from IV to oral antibiotics, and sent home with a prescription for four more days of cefdinir tablets per the ID team's recommendations to complete a total 10-day course of antibiotic treatment.

## Discussion

VGS are a group of commensal bacteria found in the oropharynx, respiratory tract, gastrointestinal tract, and genitourinary tracts. They are facultatively anaerobic, catalase-negative Gram-positive cocci that are mainly alpha-hemolytic and grow on blood agar and other non-selective solid media [4]. Though historically considered to have low pathogenicity, VGS have been documented to cause native-valve endocarditis particularly in patients with underlying valvular disorders, or bacteremia, as seen in our patient [1,5].

While there have been documented cases of VGS bacteremia secondary to other infectious sources in immunocompetent patients, the risk factors contributing to these cases versus those in immunocompromised populations are still unclear. For instance, in a 2017 National Institutes of Health case-control study, no tested clinical factors including age, gender, or recent oral procedures were noted to have an association with non-neutropenic patients [1]. It would be prudent to further examine risk factors moving forward to decrease its incidence as well as the incidence of possible sequelae such as VGS infective endocarditis.

Additionally, the clinical presentation of VGS bacteremia in adults varies depending on the source of the infection, as described in Table 2.

**Table 2:** Sources of viridans group streptococci (VGS) bacteremia and associated species As per the literature review, there are specific species within the viridans group that are more often associated with VGS bacteremia, depending on the location of the original source of infection [6].

Source of VGS bacteremia	Symptoms	VGS species
Hepatobiliary infections, abdominal abscesses, diverticulitis	Fever, abdominal pain, abdominal tenderness, possible jaundice	*S. anginosus* group (SAG): *S. anginosus* more frequent compared to *S. intermedius, S. constellatus*
Cranial infection	Headache, fever, neck pain	SAG: *S. intermedius*
Oropharynx infection	Odynophagia, dysphagia	S. mitis, S. salivarius, S. sanguinis
Upper respiratory infection	Cough, shortness of breath	SAG: *S. constellatus*

The *S. anginosus* group (SAG), a subgroup of VGS, has been frequently associated with infections of the upper respiratory and gastrointestinal tracts. SAG consists of three species, including *S. anginosus*, *S. constellatus*, and *S. intermedius*. All three of these species can result in SAG bacteremia [6]. SAG bacteremia from these species, particularly, the titular *S. anginosus*, occurs most often via translocation from hepatobiliary infections, as per various global retrospective studies [6-9]. In each of these retrospective studies, hepatobiliary infections such as ascending cholangitis were the source of approximately one-third of the cases of SAG bacteremia studied, while other sources included abdominal abscesses, diverticulitis, or malignancies. Despite reported SAG bacteremia from hepatobiliary sources including ascending cholangitis, it should be noted that there have been no previously documented occurrences in *non-neutropenic* patients specifically such as ours, particularly with an initial source of choledocholithiasis. A relevant and worthy next step for researching sources of VGS bacteremia would include further analysis of the various sources listed in Table 2, with specific sub-group analysis of prevalence in neutropenic versus non-neutropenic patients, for greater awareness of risk factors and management.

Regarding the treatment options for VGS bacteremia, one must consider susceptibilities when choosing appropriate antibiotics, as well as the type of infection being treated. In a national study of antibiotic susceptibility of VGS in the United States from 2010 to 2020, non-speciated VGS isolates, as seen in our patient, had sustained susceptibility rates of over 90% to ceftriaxone, meropenem, levofloxacin, and vancomycin, with notable resistance to clindamycin [10]. Our patient was treated appropriately with the chosen antibiotics, though it should be noted that piperacillin-tazobactam, which our patient initially received for a few days as broad-spectrum coverage prior to further speciation, was not separately tested in the study and likely warrants further investigation of up-to-date resistance rates.

Lastly, full treatment of bacteremia requires not only antibiotic therapy, but also adequate source control to prevent recurrence. In our patient, choledocholithiasis was identified prior to the bacteremia, and gastroenterologic intervention was performed to achieve source control. In patients who present with sepsis secondary to VGS bacteremia without an obvious source, it is important to consider not only oral sources, but also hepatobiliary infections or intra-abdominal abscesses, particularly when they present with abdominal pain, nausea, vomiting, or other GI symptoms as our patient did.

## Conclusions

VGS bacteremia is a well-documented clinical scenario in neutropenic patients, or patients with recent dental procedures. It is crucial to recognize that VGS bacteremia can also occur in non-neutropenic patients secondary to translocation from other sources such as respiratory or intra-abdominal infections, including this novel case where the source was choledocholithiasis. Clinicians must consider rare sources of VGS bacteremia so that adequate source control and antibiotic therapy can be rapidly implemented to reduce risk of mortality.
